# Frequency and Associated Factors of Clotted and Hemolyzed Samples in South Gonder Hospitals, Ethiopia: A Multicenter Cross‐Sectional Study, 2023

**DOI:** 10.1002/jcla.70148

**Published:** 2025-12-27

**Authors:** Birhanemaskal Malkamu, Getaneh Atikilt Yemata, Andargachew Almaw, Ayenew Assefa, Birhanu Getie, Teklehaimanot Kiros, Mulat Erkihun, Shewaneh Damtie, Tegenaw Tiruneh, Berhanu Abebaw Mekonnen, Meron Asmamaw Alemayehu, Abraham Teym, Abathun Temesgen, Gashaw Melkie Bayeh, Almaw Genet Yeshiwas, Rahel Mulatie Anteneh, Melkamu Aderajew Zemene, Tesfaneh Shimels, Chalachew Yenew, Wolde Melese Ayele, Ahmed Fentaw Ahmed, Assefa Andargie Kassa, Tilahun Degu Tsega, Chalachew Abiyu Ayalew, Sintayehu Simie Tsega, Zeamanuel Anteneh Yigzaw, Amare Genetu Ejigu, Wondimnew Desalegn Addis, Getasew Yirdaw, Kalaab Esubalew Sharew, Daniel Adane, Samuel Berihun Dagnew, Gebyaw Arega, Habitamu Mekonen

**Affiliations:** ^1^ Department of Medical Laboratory Sciences, College of Health Sciences Debre Tabor University Debre Tabor Ethiopia; ^2^ Department of Public Health, College of Health Science Debre Tabor University Debre Tabor Ethiopia; ^3^ Department of Nutrition and Dietetics, School of Public Health, College of Medicine and Health Sciences Bahir Dar University Bahir Dar Ethiopia; ^4^ Department of Epidemiology and Biostatistics, Institute of Public Health, College of Medicine and Health Sciences University of Gondar Gondar Ethiopia; ^5^ Department of Environmental Health, College of Health Sciences Debre Markos University Debre Markos Ethiopia; ^6^ Department of Environmental Health, College of Medicine and Health Science Injibara University Injibara Ethiopia; ^7^ Department of Environmental Health Sciences, Public Health, College of Health Sciences Debre Tabor University Debre Tabor Ethiopia; ^8^ Jockey Club College of Veterinary Medicine and Life Sciences City University of Hong Kong Hong Kong SAR China; ^9^ Department of Public Health, College of Medicine and Health Sciences Injibara University Injibara Ethiopia; ^10^ Department of Epidemiology School of public health Cheeloo College of Medicine Shandong University Jinan China; ^11^ Department of Medical Nursing, School of Nursing, College of Medicine and Health Science University of Gondar Gondar Ethiopia; ^12^ Department of Health Promotion and Behavioral Sciences, School of Public Health, College of Medicine and Health Sciences Bahir Dar University Bahir Dar Ethiopia; ^13^ Department of Midwifery, College of Medicine and Health Sciences Injibara University Injibara Ethiopia; ^14^ Department of Environmental Health Science, College of Medicine and Health Sciences Debre Markos University Debre Tabor Ethiopia; ^15^ Department of Medicine, College of Medicine and Health Sciences Injibara University Injibara Ethiopia; ^16^ Department of Clinical Pharmacy, College of Health Sciences Debre Tabor University Debre Tabor Ethiopia; ^17^ School of Medical Laboratory Sciences, Faculty of Health Sciences Jimma University Jimma Ethiopia; ^18^ Department of Human Nutrition Debre Markos University Debre Markos Amhara Ethiopia

**Keywords:** clotting, error, hematology, hemolysis, pre‐analytical, RBC

## Abstract

**Background:**

Accurate hematological analysis relies heavily on the integrity of blood samples, which can be compromised by pre‐analytical errors such as hemolysis and clotting. This study aimed to determine the frequency and associated factors of clotted and hemolyzed samples in selected hospitals in South Gonder, Ethiopia.

**Methods:**

This institutionally based cross‐sectional study was conducted from September to December 2023 in northwestern Ethiopia at the medical hematology laboratories of selected South Gonder Zone hospitals. Debre Tabor's specialized referral hospital was selected. Addis Zemen and Nefas Mewcha primary hospitals were chosen at random.

**Results:**

Among the 2331 test samples, 829 (35.6%) were clotted, and 269 (11.5%) were hemolyzed. We found a significant association of clotting with contamination, inadequate sample volume, puncture site other than the median cubital region, more than three attempts to collect blood, and the use of a partially filled collection tube when attempting another vein puncture.

**Conclusion:**

On the basis of our observations, the findings presented here have a significant impact on patient diagnosis and treatment, leading to delayed or inaccurate diagnoses, inappropriate treatment decisions, increased risk of adverse events, and increased healthcare costs.

## Graphical abstract

This study, conducted in South Gonder hospitals in Ethiopia, investigated the prevalence and causes of clotted and hemolyzed blood samples submitted for hematological testing. The findings revealed a high rate of sample quality issues, with significant associations identified between pre‐analytical factors like collection technique and sample integrity. The conclusion emphasizes the substantial impact of these compromised samples on patient care, potentially leading to diagnostic errors and adverse outcomes.

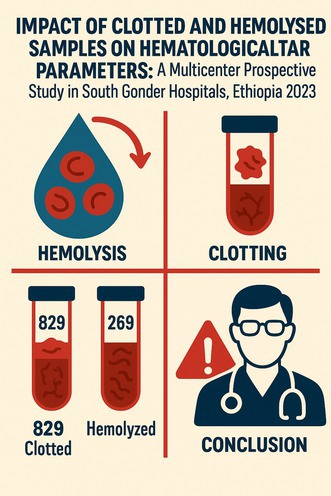

**Abbreviations**
AOR: adjusted odds ratio
CI: confidence interval
COR: crude odds ratio
DTCSH: Debre Tabor Comprehensive Specialized Hospital
EDTA: ethylene diamine tetra acetic acid
ESR: erythrocyte sedimentation rate
IRB: institutional review board

## 1 Introduction

Accurate hematological analysis relies heavily on the integrity of blood samples, which can be compromised by hemolysis and clotting. High‐quality medical diagnostics are essential to provide patients with dependable medical care. Among other clinical specialties, laboratory medicine is critical for patient safety [1]. Red blood cells undergo changes and injury during processing and storage, leading to clotting or hemolysis [2].

Hemolysis is the breakdown or rupture of the red blood cell/erythrocyte cell membrane, which releases intracellular components in plasma or serum. It can be caused by diseases such as hemolytic anemia or centrifugation [2, 3, 4].

Visual inspection is the only method suitable for routine monitoring of all RBCs currently available at blood banks and hospitals to determine hemolysis. This method, in contrast with Hb quantification methods (Harboe, Drabkin's, or automated analyzers), is highly inaccurate. Consequently [5, 6], hemolytic RBCs could be transfused into patients or analyzed for hematological tests, or some RBCs could be unnecessarily destroyed [7].

Clotting, on the other hand, is a normal physiological response to prevent blood loss. However, in laboratory and transfusion settings, clotting must be avoided to maintain sample integrity. The coagulation process involves the activation of thrombin and other clotting factors, which are tightly regulated by inhibitors such as anti‐thrombin III and tissue factor pathway inhibitors. These mechanisms prevent excessive clot formation, reducing the risk of complications such as thrombosis [8].

Blood and vascular cells, cofactors, inhibitors, and plasma‐derived enzymes interact to form the enzymatic‐blood coagulation process called clotting. The platelet offers locations for the expression and assembly of catalysts. These stoichiometric protein inhibitors play crucial roles in maintaining balance within the coagulation process by preventing excessive clot formation [2, 6, 9, 10]. Their action ensures that while pro‐coagulant mechanisms are activated, the body can still regulate and limit coagulation to avoid complications such as thrombosis.

Anticoagulants such as heparin, EDTA, and sodium citrate are essential for preventing clotting in vitro, preserving blood in a state suitable for hematological tests or transfusion. Proper handling and storage of blood samples are critical to ensuring diagnostic accuracy and patient safety [11].

Despite the global recognition of pre‐analytical errors as a major contributor to laboratory inaccuracies, there is a significant gap in data from Ethiopia, particularly concerning the frequency and contributing factors of sample rejection. To our knowledge, no comprehensive multicenter cross‐sectional study has been conducted in the South Gonder Hospitals to quantify and analyze sample rejection due to clotting and hemolysis. This study was therefore undertaken to assess the prevalence of and identify the key factors associated with clotted and hemolyzed samples, providing crucial baseline data to inform quality improvement strategies in this region. By addressing local procedural and supply factors, our findings add novel knowledge and provide crucial baseline data for improving laboratory quality in this regional context.

## 2 Methods and Materials

### 2.1 Study Design, Area, and Period

An institutionally based cross‐sectional study was conducted from September to December 2023 in North West Ethiopia in the medical hematology laboratories of selected South Gondar Zone Hospitals.

### 2.2 Study Participants

All blood samples were collected during working hours in the hematology laboratories of the three selected hospitals during the study period and were subsequently observed. On the other hand, tests of blood samples collected on weekends, during lunch breaks, and during night shifts were excluded.

### 2.3 Sampling Method

The sample size was determined via the single population proportion formula,

$$n=\mathrm{Z\alpha}/22\;p\times \left(1-p\right)/\text{d2}.$$

We assumed a 95% confidence level, a 2% margin of error, and a 50% proportion of samples with hemolysis and clotting. The final determined sample size was 2401.

In the South Gonder Zone, there are nine hospitals (one comprehensive specialized referral and eight primary hospitals). Debre Tabor's Comprehensive Specialized Hospital was selected purposefully since it is the sole Comprehensive Specialized hospital in the South Gonder Zone and provides tertiary health care by Ethiopian health tiers. Addis Zemen and Nefas Mewcha primary hospitals were chosen at random from the eight primary hospitals on the basis of the homogeneity of the health care delivery system, as they are all primary‐level health care facilities under Ethiopia's health tier system.

On the basis of the flow of samples to the hematology lab (using the formula ni = [*n*/*N*] Ni), the sample size was allocated proportionally to the three hospitals: 1078 for Debre Tabor specialized referral hospital, 603 for Nefas Mewcha, and 720 for Addis Zemen hospital.

Since ni is the sample size for each hospital, n is the total sample size from three hospitals, which was 2401; *N* is the flow of samples to hematology in the three hospitals, which was taken from the last 3 months' registration records, and Ni is the expected number of samples that came to the hematology laboratory of each hospital, which was predicted from the last 3 months; Ni for DCSRH was 3234; Ni for Addis Zemen Hospital was 2160, Ni for Nefas Mewucha Hospital was 1809; and N, which is the total number of samples that came to the hematology laboratory, was 7203.

During the study period, a systematic random sampling technique was used to pick study units from samples that came to the hematology laboratory. The Kth interval (*K* = 3) was estimated via a list of samples that came to the hematology laboratory. The lottery approach was used to determine the first random start, and every Kth interval from that point was chosen until the final sample was completed.

### 2.4 Inclusion/Exclusion Criteria

Samples collected during weekends, lunch breaks, and night shifts were excluded from this study to ensure consistent data collection from regular, day‐to‐day operations and to minimize the potential for bias associated with varying staff availability and workload.

### 2.5 Data Collection and Sample Processing

#### 2.5.1 Data Quality Assurance and Management

Clotted and hemolyzed samples were identified via visual inspection by trained laboratory personnel. Information regarding the factors associated with clotting and hemolysis was collected from the medical hematology laboratories of the selected hospitals using a pretested questionnaire and a checklist.

Clotted and hemolyzed samples were assessed and defined by visual inspection. A sample was considered clotted if it contained any fibrin clots, micro‐clots, or gel‐like consistency upon gentle mixing. Hemolysis was defined as the visible pink‐to‐red discoloration of the plasma or serum, indicating the rupture of red blood cells. The criteria for defining a sample as clotted or hemolyzed were standardized across all participating laboratories to ensure consistency.

To ensure the quality of the data, the data collection tool was assessed by professionals for appropriateness and overall evaluation. A pretest was performed on 138 samples at Bahirdar University Tibebe Gion Specialized Hospital from September 20 to September 28, 2023.

After the questionnaire was completed, it was checked by the researcher, and some modifications to the questionnaire were made for unclear and difficult questions. These pretest data were not included in the analysis of this study. Training was given for four laboratory technician data collectors by the principal investigator.

The completeness of the questionnaire and checklist was assessed at the end of each day by the principal investigator. To ensure the quality of the data, the questionnaire was first tested by colleagues to avoid any ambiguity in the questions. Daily supervision was conducted during the data collection period.

#### 2.5.2 Data Processing and Statistical Analysis

The data were entered into Epi‐data software (Version: 3.0.4), cleaned, and exported into IBM Corp.'s Statistical Package for Social Science version 20 software (IBM Corp., Armonk, NY, USA) for analysis. Descriptive statistics such as frequencies and percentages were used to summarize the data. The data are presented in figures and tables. Binary logistic regression was performed, and variables with a *p*‐value of < 0.25 were fitted into the multivariable logistic regression analysis. The strength of the associations between predictors and outcomes was calculated via the crude odds ratio (COR) and adjusted odds ratio (AOR) with a 95% CI. In all the cases, a P‐value of less than 0.05 was considered statistically significant.

The overall fit of the final multivariable model was assessed using the Hosmer‐Lemeshow test, which indicated a good fit (*χ* ^2^ (8) = 14.186, *p* = 0.077). The model explained a small but significant proportion of the variance in the outcome, as indicated by a Nagelkerke's *R* ^2^ value of 0.044.

Multicollinearity among predictors was assessed by examining the standard errors of the coefficients and a correlation matrix. No evidence of significant multicollinearity was detected, with all variables showing minimal correlation and standard errors within an acceptable range.

### 2.6 Ethical Consideration

Ethical clearance for this was obtained from the institutional review board (IRB) of the Institute of Health, Jimma University (Ref. No. IHRPGH/449). Before data collection, informed consent was obtained from all participants. For any participant under the age of 18, informed consent was obtained from their legal guardian. After ethical approval was received, permission to conduct the study was obtained from the head of the School of Medical Laboratory Science and the chief clinical director of the DTCSH. A support letter from the Jimma University Health Science Research Coordinating Office was written to DTCSH. During data collection, only the code of the sample was used, and the privacy and identity of the research data were confidential. Patient confidentiality was strictly maintained throughout the study. All data were anonymized, using a unique code for each sample, and no personally identifiable information was collected or linked to the results. The data were stored in a password‐protected computer, and access was limited to the research team to ensure data security.

## 3 Results

### 3.1 Prevalence of Clotted and Hemolyzed Samples in DTCSH

**FIGURE 1:**
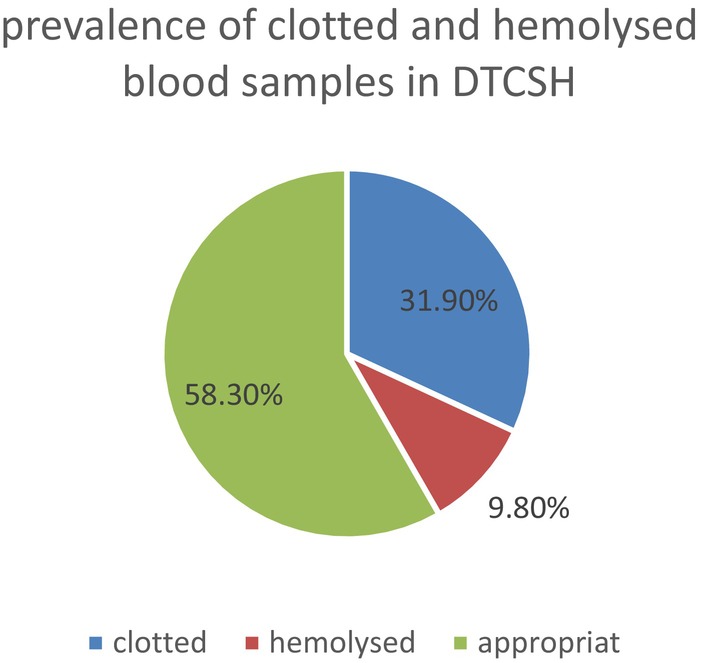
Prevalence of clotted and hemolyzed samples in Debre Tabor Comprehensive Specialized Hospital (DTCSH), 2023.

In this study, among the 1032 samples from Debre Tabor Comprehensive Specialized Hospital (DTCSH), we observed 329 (31.9%) clotted samples and 101 (9.8%) hemolyzed samples (Figure 1).

### 3.2 Prevalence of Clotted and Hemolyzed Samples in Addis Zemen Hospital

**FIGURE 2:**
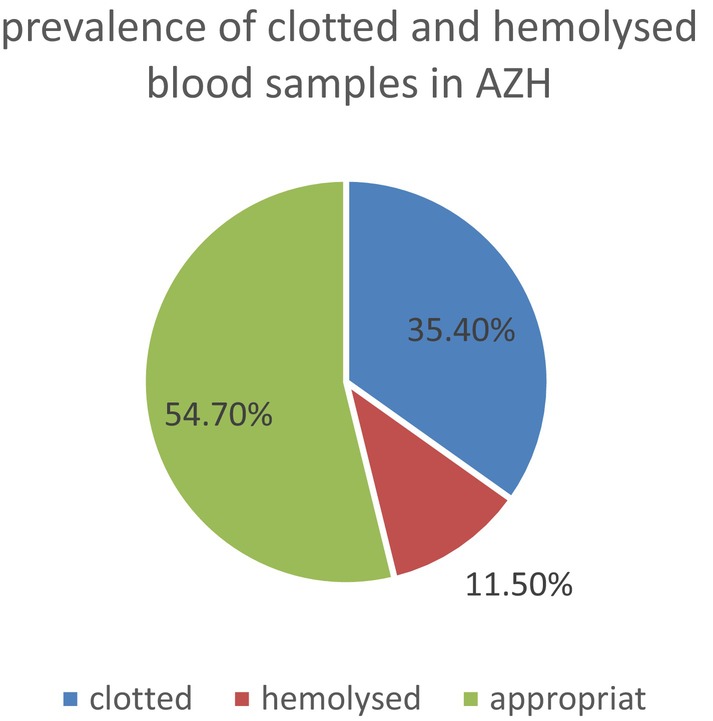
Prevalence of clotted and hemolyzed samples in Addis Zemen Hospital (AZH), 2023.

In this study, among the 707 samples from Addis Zemen Hospital (AZH), we observed 250 (35.4%) clotted samples and 81 (11.5%) hemolyzed samples (Figure 2).

### 3.3 Prevalence of Clotted and Hemolyzed Samples in Nefas Mewcha Hospital

**FIGURE 3:**
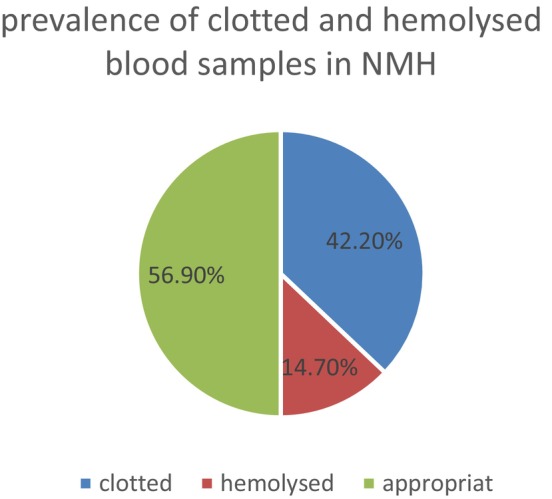
Prevalence of clotted and hemolyzed blood samples in Nefas Mewcha Hospital (NMH), 2023.

In this study, among the 592 samples from Nefas Mewcha Hospital (NMH), we observed 250 (42.2%) clotted samples and 87 (14.7%) hemolyzed samples (Figure 3).

Prevalence of Hemolysis in selected South Gondar Zone Hospitals Hemolysis was observed in 269 (11.5%) of the 2331 samples.

### 3.4 Factors Associated With Sample Clotting and Hemolysis

In our study, several factors were associated with blood sample clotting. These included using a collection tube contaminated with blood (AOR = 1.41, 95% CI = 1.12–1.79), choosing a puncture site other than the median cubital vein (AOR = 1.46, 95% CI = 1.01–2.11), requiring more than three attempts to collect blood (AOR = 1.53, 95% CI = 1.03–2.28), using a partially filled tube when attempting another vein puncture (AOR = 1.296, 95% CI = 1.01–1.68), and the blood draw occurring during the afternoon shift (AOR = 1.329, 95% CI = 1.12–1.59). Conversely, an inadequate sample volume (less than one‐third of the collection tube) was associated with a decreased risk of clotting (AOR = 0.77, 95% CI = 0.65–0.93) (Table 1).

**TABLE 1:** COR and AOR results of factors associated with clotting of hematology test samples.

Study variable	Category	Clotting		COR, 95% CI	*p*	AOR, 95% CI	*p*
		Yes	No				
Collection tube contaminated with blood	Yes	154 (18.6%)	350 (23.3%)	1.33 (1.08–1.65)	0.008	1.41 (1.12–1.79)	0.003*
	No	675 (81.4%)	1152 (76.7%)	1			
Sample inadequate volume (< 1/3 of the collection tube)	Yes	541 (65.3%)	873 (58.1%)	0.74 (0.62–0.88)	0.001	0.77 (0.65–0.93)	0.006*
	No	288 (34.7%)	629 (41.9%)	1			
Puncture site other than median cubital	Yes	277 (33.4%)	497 (33.1%)	1.24 (1.003–1.54)	0.047	1.46 (1.01–2.11)	0.045*
	No	286 (34.5%)	621 (41.3%)	1			
More than 3 attempted to collect blood	Yes	20 (30.3%)	46 (69.7%)	1.27 (1.11–1.43)	0.067	1.53 (1.03–2.28)	0.035*
	No	599 (33.9%)	1168 (66.1%)	1			
Using a partially filled collection tube when attempting another vein puncture	Yes	255 (16.3%)	412 (14.0%)	0.61 (0.71–1.22)	0.614	1.296 (1.01–1.68)	0.004*
	No	574 (54.8%)	1090 (59.2%)	1			
Shift of the day	Morning	477 (57.5%)	722 (48.1%)	1			
	Afternoon	352 (42.5%)	780 (51.9%)	1.46 (1.23–1.74)	0.001	1.329 (1.12–1.59)	0.001*
Pressing the syringe	Yes	276 (30.8%)	619 (69.2%)	1.40 (1.18–1.68)	0.001	1.14 (0.801–1.63)	0.46
	No	553 (38.5%)	883 (61.5%)	1			

* *p* < 0.05.

In this study, an inappropriate sample‐to‐anticoagulant ratio (AOR = 1.47, 95% CI = 1.12–1.94) and underfiling of collection tubes (less than 90% full; AOR = 2.58, 95% CI = 1.52–4.36) were associated with hemolysis (Table 2).

**TABLE 2:** COR and AOR results of factors associated with hemolysis of hematology test samples.

Study variable	Category	Hemolyzed		COR, 95% CI	*p*	AOR, 95% CI	*p*
		Yes	No				
Inappropriate sample to anticoagulant ratio	Yes	163 (10.6%)	1380 (89.4%)	1.32 (1.01–1.71)	0.04	1.47 (1.12–1.94)	0.005*
	No	106 (13.5%)	682 (86.5%)	1			1
Under filing of collection tubes (< 90% filling of collection tubes)	Yes	217 (12.3)	1504 (87.7%)	1.5 (1.02–2.19)	0.037	2.58 (1.52–4.36)	0.001*
	No	52 (6.6%)	558 (93.4%)	1			1
Shift of the day	Morning	132 (11.0%)	1067 (89.0%)	1.11 (0.86–1.43)	0.41	1.10 (0.86–1.43)	0.43
	Afternoon	137 (12.1%)	995 (87.9%)	1			1
Pressing the syringe	Yes	148 (11.1%)	1078 (88.9%)	1.09 (1.01–1.19)	0.04	1.36 (1.02–1.70)	0.17
	No	121 (9.4%)	964 (90.6%)	1			

* *p* < 0.05.

## 4 Discussion

### 4.1 Introduction to Findings

The findings of this study provide critical new data on the prevalence and factors associated with pre‐analytical errors in the South Gonder region, an area where such comprehensive, multicenter data were previously unavailable. By identifying specific issues such as improper handling and inadequate volume, our research serves as a foundational step for targeted quality improvement initiatives in local healthcare settings.

Hemolysis and clotting of blood samples are well‐recognized challenges in laboratory medicine that can significantly compromise the accuracy of diagnostic test results. These sample quality issues can lead to erroneous measurements, particularly for hematological parameters such as hemoglobin levels, platelet counts, and white blood cell analysis. As a result, incorrect or unreliable data may impact clinical decision‐making and patient care. Understanding the extent to which hemolysis and clotting affect hematological results is crucial, as it allows for the development of improved protocols for blood collection, handling, and analysis, ultimately enhancing the reliability and consistency of laboratory diagnostics.

### 4.2 Comparison With Literature

In this study, from the observed overall samples collected for tests in the hematology laboratory from September to December, 829 (35.6%) clotted samples were observed. Our findings were lower than those of another study performed in southern Brazil, University Hospital de Clínicas de Porto Alegre, in 2012, which was 43.8% [14]. However, our findings were greater than those of studies performed in the neonatal intensive care unit of a hospital in Qatar: 4.8% in 2017 and 2018; 2.4% in 2019 [15], and another study performed in the hematology laboratory of a tertiary care hospital in South India reported a clotted sample frequency of 0.12% [16]. The possible reasons for this discrepancy might be blood collection procedures, differences in sample size, differences in laboratory equipment and techniques, staff performance related to workload, patient volume, resource availability, training and experience, and the prevalence of underlying conditions that may contribute to clotting.

On the other hand, the percentage of hemolyzed samples in our study was 269 (11.5%). This finding was lower than that of another study performed in Ethiopia at Debre Markos Referral Hospital, which reported a hemolysis frequency of 21.6% [17], and another study performed in southern Brazil, University Hospital de Clínicas de Porto Alegre, which included 17.9% hemolyzed samples [14]. This difference may be due to differences in sample size, staff performance related to test sample handling such as vigorous sample handling and prolonged tourniquet application; and the presence of primary factors (such as sickle cell anaemia and thalassemia) that may subsidize hemolysis.

### 4.3 Factors Associated With Clotting and Hemolysis

In this study, the odds of clotting in contaminated blood collection tubes were 1.41 times greater than those in non‐contaminated collection tubes. Venipuncture at a site other than the median cubital vein increased the odds of clotting by 1.46 times compared with venipuncture at the median cubital vein. The odds of clotting were 1.53 times greater when more than three venipuncture attempts were needed. Using a partially filled collection tube (less than one‐third of the collection tube) during subsequent venipuncture attempts was associated with a 1.29‐fold greater odds of clotting. Blood samples collected during the afternoon shift (defined as 13:30–17:00) presented a 1.33‐fold greater odds of clotting. Conversely, inadequate sample volume (less than one‐third of the collection tube) was associated with lower odds of clotting (OR = 0.77).

The finding that inadequate sample volume was associated with a lower odds of clotting (AOR = 0.77). While standard practice emphasizes adequate sample‐to‐anticoagulant ratios to prevent clotting, this finding may reflect a complex interplay of factors specific to our study. One possible explanation is that samples with very small volumes may have been collected from difficult veins or from patients with underlying conditions that affect blood flow, leading to more cautious handling by phlebotomists. Alternatively, it is plausible that the small volume of blood in these tubes did not contain enough pro‐coagulant factors to initiate and form a visible clot. This result underscores the need for further investigation and highlights the possibility of confounding variables not captured in our analysis. We believe this finding warrants cautious interpretation within the context of our study's limitations.

Conversely, the odds of hemolysis were 1.47 times greater in samples with an inappropriate sample‐to‐anticoagulant ratio. Underfilling collection tubes (< 90% full) increased the odds of hemolysis by 2.58 times.

The high prevalence of pre‐analytical errors identified in this study (35.6% clotting and 11.5% hemolysis) has significant implications for patient care and healthcare system efficiency. While this study did not quantify the financial and time‐related costs associated with these errors, their impact is substantial. Sample rejection leads to repeat venipuncture, causing patient discomfort and anxiety, and delaying diagnosis and the start of treatment. Furthermore, the need for re‐collection and re‐processing of samples results in a significant waste of valuable staff time, laboratory resources, and reagents. We therefore highlight the need for future studies to quantify the economic burden and time wastage resulting from these errors to build a stronger case for resource allocation toward quality improvement initiatives.

## 5 Recommendation

To address these issues, we recommend improvements in several areas. First, enhancing data management in health services is essential. This includes developing a robust system for tracking and managing blood samples, including but not specific to training on proper venipuncture techniques, correct collection tube filling, and blood transfer procedures; implementing electronic data capture systems to minimize data entry errors; and standardizing blood collection procedures. Additionally, providing adequate staff training and resources, implementing quality control measures to monitor sample quality, and utilizing automated blood collection systems are crucial. We also suggest conducting a larger study with a more diverse population to increase the generalizability of the findings. Second, laboratory procedures should be improved by regularly inspecting and maintaining laboratory equipment, ensuring adequate staffing levels, and managing the workload of laboratory personnel appropriately. Moreover, a system for timely sample processing should be implemented to minimize delays.

## 6 Study Limitation

We would like to acknowledge several limitations of the study, including the relatively short study period, the inability to include data on comorbidities and medication use, which could influence sample quality, and the lack of information on specific laboratory procedures and equipment. Another limitation of this study is the lack of information on the specific quality control and calibration procedures used by the participating laboratories. While all laboratories are expected to follow standard protocols, this information was not collected, and its potential impact on the data could not be assessed.

This study did not collect data on specific patient‐related confounders, such as underlying comorbidities (e.g., sickle cell anemia, thalassemia) or medication use (e.g., anticoagulants), which could directly influence blood sample quality. The absence of this information represents a limitation, as these factors may have a direct impact on the prevalence of clotting and hemolysis. Additionally, the exclusion of samples collected during weekends, night shifts, and lunch breaks may introduce a selection bias, as a different set of factors (e.g., lower staffing, emergency cases) might influence sample quality during these periods.

## 7 Conclusion

In conclusion, this multicenter prospective study revealed a high prevalence of pre‐analytical errors, with clotting and hemolysis rates of 35.6% and 11.5%, respectively, in the South Gonder Hospitals. The study identified several contributing factors, including inadequate sample volume and improper handling of samples. Our findings highlight that pre‐analytical errors remain a significant challenge to the quality of clinical laboratory analysis in this region.

## Author Contributions

Conceptualization and design of the study: Birhanemaskal Malkamu and Getaneh Atikilt Yemata. Acquisition of data: Meron Asmamaw Alemayehu, Abraham Teym, Abathun Temesgen, Gashaw Melkie Bayeh, Almaw Genet Yeshiwas, Rahel Mulatie Anteneh, Melkamu Aderajew Zemene, Berhanu Abebaw Mekonnen, and Tesfaneh Shimels. Analysis and interpretation of data: Birhanemaskal Malkamu, Getaneh Atikilt Yemata, Andargachew Almaw, Ayenew Assefa, Abraham Teym, Abathun Temesgen, Birhanu Getie, Getasew Yirdaw, Teklehaimanot Kiros, Mulat Erkihun, Shewaneh Damtie, and Tegenaw Tiruneh. Investigation: Birhanemaskal Malkamu, Chalachew Yenew, Wolde Melese Ayele, Ahmed Fentaw Ahmed, Assefa Andargie Kassa, Tilahun Degu Tsega, Sintayehu Simie Tsega, Zeamanuel Anteneh Yigzaw, Amare Genetu Ejigu, Wondimnew Desalegn Addis, Getasew Yirdaw, and Kalaab Esubalew Sharew. Methodology: Birhanemaskal Malkamu, Getaneh Atikilt Yemata, Daniel Adane, Habitamu Mekonen, Meron Asmamaw Alemayehu, and Teklehaimanot Kiros. Project administration: Birhanemaskal Malkamu, Getaneh Atikilt Yemata, Habitamu Mekonen, Abraham Teym, Abathun Temesgen, and Getasew Yirdaw. Supervision: Birhanemaskal Malkamu, Getaneh Atikilt Yemata, Habitamu Mekonen, Meron Asmamaw Alemayehu. Validation: Birhanemaskal Malkamu, Getaneh Atikilt Yemata, Andargachew Almaw, Gashaw Melkie Bayeh, Rahel Mulatie Anteneh, and Melkamu Aderajew Zemene. Drafting the article: Birhanemaskal Malkamu, Getaneh Atikilt Yemata, Berhanu Abebaw Mekonnen, Tesfaneh Shimels, Ayenew Assefa, and Abraham Teym, Abathun Temesgen. Writing, reviewing, and editing: Birhanemaskal Malkamu, Habitamu Mekonen, Daniel Adane, Getasew Yirdaw, and Getasew Yirdaw. Final approval: Birhanemaskal Malkamu, Getaneh Atikilt Yemata, and Habitamu Mekonen.

## Funding

The authors have nothing to report.

## Data Availability

All the data on which the conclusions of this manuscript were drawn are available from the corresponding author. So, anyone who needs the data can get it upon reasonable request.
